# Undergraduate psychiatric education in South Asian countries

**DOI:** 10.4103/0019-5545.37314

**Published:** 2007

**Authors:** J. K. Trivedi, Mohan Dhyani

**Affiliations:** Department of Psychiatry, King George Medical University, Lucknow - 226 003, Uttar Pradesh, India

Asia has some of the largest conglomerations of human populations and also the fastest-growing economies of the world. About 25% of the world's population lives in the South Asian region, and one-fifth of the psychiatrically ill in the world live in this region. The mental-health manpower to cater to this huge population is grossly inadequate. The government expenditure on mental health in majority of the SAARC countries is <1% of the total national health budget.[1]

## SHORTAGE OF MENTAL-HEALTH PROFESSIONALS

There is a shortage of “skilled health workforce” in various fields, which includes mental-health services, which are facing a deficiency worldwide. This shortage is worst in the deprived countries with already limited resources. WHO notes that developing nations like India are not training enough psychiatrists. India has only 3,000 psychiatrists for 110 million people.[2] Mental-health services are poorly developed, not only due to poor resource allocation but also due to general ignorance of ‘mental health’-related issues at all levels of government planning and implementation. The situation is similar in other South Asian countries.

The World Health Organization's annual report for 2006[3] focuses on this issue. It contains an expert assessment of the current crisis in global health workforce. The report lays out a 10-year action plan focusing on “global partnerships.”

Recommendations of the 59^th^ World Health Assembly, 27^th^ May 2006, include:

1. Rapid scaling up of health workforce production
2. More direct investment in the training and support of health workers
3. Promoting “training partnerships” between industrialized and developing countries

## PSYCHIATRY TRAINING AT UNDERGRADUATE LEVEL: GROWING IMPORTANCE

In most of the South Asian countries, the psychiatrist alone will not be able to cater to the mental-health needs of the population. Most of the patients first contact the physician for treatment. Unfortunately due to a deficient training at the undergraduate level, most doctors are unable to recognize and correctly treat psychiatric disorders. The basic purpose of teaching and training at the undergraduate level is to prepare medical graduates to serve better at primary health-care level. The patients with physical illness have concomitant emotional problems requiring professional handling; therefore, teaching of Psychiatry at the undergraduate level becomes even more relevant and essential. Teaching Psychiatry at the undergraduate level can have a positive impact on medical students and their attitudes to mental illness, reducing stereotyping and increasing empathy.[4]

Emphasis on training in Psychiatry during undergraduate training is dismally low; the Medical Council of India guidelines show that students are required to participate only in a two-week program of clinical postings, excluding a number of theory lectures. In many of the colleges the department of Psychiatry has come into existence recently, and the faculty teaching the undergraduates is relatively junior and not fully experienced in teaching undergraduates.[5]

All levels of training in Psychiatry to undergraduates and postgraduates lack a competence-based curriculum. This can be rectified only if policy and decision makers recognize the importance of Psychiatry, not only in medical education but also in primary health care. Unfortunately, the representation of psychiatrists at the higher levels (the MCI, university senates and university syndicates) is marginal. Furthermore, Psychiatry has a very thin slice of the medical curriculum “cake.” The situation is reflected similarly in the other South Asian countries. Sri Lanka is an exception, and most colleges have eight or more weeks of training in psychiatry; and also in most medical colleges, Psychiatry is one of the subjects in the final exams.[6]

It is an unfortunate paradox that the countries in which there are least resources for Psychiatry teaching are generally those in which the need is the greatest. This problem is bound to grow with the increased prevalence of psychiatric diseases.

## PSYCHIATRY TRAINING FOR UNDERGRADUATES: THE PRESENT STATE

Despite more than half a century of phenomenal scientific progress in managing the mentally ill, age-old fears die hard; ignorance flourishes, takes root and spawns rigid prejudice among those concerned with mental health. The public's prejudice results from a sordid state of mental-health services and facilities. This leads to political policy-making based on prejudice perpetrated by those in positions of power.

The basic problem is that students can feel and empathize for the physically ill, but they are overwhelmed by fear when they see the mentally ill. At organizational level, many medical schools even today do not have independent departments of Psychiatry, and Psychiatry is catered for within the department of Medicine. For the schools that do have a Psychiatry department, the staffing situation is generally poor. Moreover, training in Psychiatry is perfunctory and tends to address the cognitive aspects rather than the psychomotor or affective aspects of mental disorder. The undergraduate curriculum of the MCI (Medical Council of India) gives meager representation to Psychiatry. Undergraduate medical students are exposed to Psychiatry for only 15-20 hours by way of didactic lectures during the entire course of their medical education, which spans 4.5 years.

The objective of undergraduate psychiatric education should be to equip medical students with core psychiatric knowledge useful in daily medical practice rather than filling curriculum time and teaching a charade that need not be examined. Students are overloaded with brain chemistry, neurotransmitters research data on psychoses but are starved of recognition of anxiety states, depression in nonpsychiatric medical settings.

This may be due to teaching undergraduate students on cases meant for postgraduates; because in psychiatric wards, units are full of patients who are severely ill and mostly psychotic. Primary-care psychiatric problems, anxiety, depression are in abundance in primary-care clinics and medical wards where Psychiatry is not taught. This leads to failure of undergraduate students to recognize common problems like anxiety and depression. The undergraduates may be taught the psychiatric manifestations of common physical disorders like delirium, which is found in abundance in medical and surgical wards.

Psychiatry is still not a separate subject in the medical curriculum in most of the countries of this region. It is still under the umbrella of General Medicine; and in examination also, it is kept with General Medicine. To blossom as a subject, Psychiatry needs to have its own space in the medical curriculum. In most of the medical schools of this region, knowledge of psychiatric illnesses is not evaluated in the examinations, psychiatric cases are not kept in clinical examination and viva voce part also seldom covers Psychiatry.

## FUTURE DIRECTIONS

To meet the acute shortage of trained psychiatrists in this region, there is a need for both short- and long-term programs. In the short term, postgraduate training programs should be instituted in all medical colleges. The quality of training and examination of the programs should be supervised by either the regional universities or, preferably, a central body. Long-term plans should focus on reforming undergraduate medical education to incorporate expanded, structured training in Psychiatry so that M.B.B.S. doctors are well prepared for the high standards of postgraduate training and examinations. In addition, Psychiatry sub-specialty training programs need to be started. However, to accomplish these ambitious goals, the countries of this region need to have clear ideas and programs to reach the desired goal. To overcome the shortage of teachers in Psychiatry in this region, a system of visiting teachers could be initiated. In Europe and America, a large number of eminent college members and fellows are of Southeast Asian origin. They could be asked to provide some teaching and training in this region. The logistics of operating such a system - by whom, for how long and how much - needs to be worked out. Short-term “exchange” teaching programs for postgraduate students among these countries for continuous medical education need to be encouraged. The future doctor should be sympathetic towards patients with psychological complaints, be able to detect mental disorders and treat simple cases and be aware when to seek a specialist's help.

## CONCLUSIONS

Proposals for the future are:

- Increase exposure to Psychiatry in undergraduate curriculum
  - Psychiatry should be a compulsory subject with university examination for the M.B.B.S. students.
  - There should be a separate Theory paper, Clinical exam and Viva voce, as for other subjects.
  - The examiners shall be from Psychiatry faculty only.
  - There should be minimum one month clinical posting and 60 lectures.
  - There should be compulsory posting in Psychiatry during internship for two to four weeks.
  - Few lectures in Psychiatry may be included in the first or second semester, for orientation.
- Psychiatry departments in all medical colleges should be strengthened with a minimum strength of 20 beds and a minimum faculty consisting of one professor, one associate professor and two asst. professors/lecturers.
